# Histone Deacetylase 1 Reduces Lipogenesis by Suppressing SREBP1 Transcription in Human Sebocyte Cell Line SZ95

**DOI:** 10.3390/ijms22094477

**Published:** 2021-04-25

**Authors:** Hye Sun Shin, Yuri Lee, Mi Hee Shin, Soo Ick Cho, Christos C. Zouboulis, Min Kyoung Kim, Dong Hun Lee, Jin Ho Chung

**Affiliations:** 1 Department of Dermatology, Seoul National University College of Medicine, Seoul 03080, Korea; shs6211@snu.ac.kr (H.S.S.); lepommier@snu.ac.kr (Y.L.); fiona1@hanmail.net (M.H.S.); chlroe@hotmail.com (S.I.C.); 2 Department of Biomedical Sciences, Graduate School, Seoul National University, Seoul 03080, Korea; 3 Medical Research Center, Institute of Human-Environment Interface Biology, Seoul National University, Seoul 03080, Korea; 4 Dessau Medical Center, Departments of Dermatology, Venereology, Allergology and Immunology, Faculty of Health Sciences Brandenburg, Brandenburg Medical School Theodor Fontane, 06847 Dessau, Germany; christos.zouboulis@klinikum-dessau.de; 5 Institute on Aging, Seoul National University, Seoul 03080, Korea

**Keywords:** histone deacetylase 1, SREBP1, lipogenesis, sebocytes, sebum, histone acetylation

## Abstract

Proper regulation of sebum production is important for maintaining skin homeostasis in humans. However, little is known about the role of epigenetic regulation in sebocyte lipogenesis. We investigated histone acetylation changes and their role in key lipogenic gene regulation during sebocyte lipogenesis using the human sebaceous gland cell line SZ95. Sebocyte lipogenesis is associated with a significant increase in histone acetylation. Treatment with anacardic acid (AA), a p300 histone acetyltransferase inhibitor, significantly decreased the lipid droplet number and the expression of key lipogenic genes, including sterol regulatory-binding protein 1 (SREBP1), fatty acid synthase (FAS), and acetyl-CoA carboxylase (ACC). In contrast, treatment with trichostatin A (TSA), a histone deacetylase (HDAC) inhibitor, increased the expression of these genes. Global HDAC enzyme activity was decreased, and HDAC1 and HDAC2 expression was downregulated during sebaceous lipogenesis. Interestingly, HDAC1 knockdown increased lipogenesis through SREBP1 induction, whereas HDAC1 overexpression decreased lipogenesis and significantly suppressed *SREBP1* promoter activity. HDAC1 and SREBP1 levels were inversely correlated in human skin sebaceous glands as demonstrated in immunofluorescence images. In conclusion, HDAC1 plays a critical role in reducing SREBP1 transcription, leading to decreased sebaceous lipogenesis. Therefore, HDAC1 activation could be an effective therapeutic strategy for skin diseases related to excessive sebum production.

## 1. Introduction

Sebaceous glands are holocrine glands concentrated in the face and scalp and located on most surfaces of the human body. Sebocytes, specialized sebaceous gland cells, produce and secrete lipids, consisting of triglycerides, fatty acids, wax esters, squalene, cholesterol esters, and cholesterol [1]. Sebaceous lipids and cell debris are constituents of sebum, which protects the skin by balancing moisture and providing innate immunity. However, excessive sebum excretion is a major factor in the pathophysiology of acne vulgaris [2].

Sebum production is dependent on *de novo* lipogenesis in sebocytes [3]. Recent studies have shown that sebaceous lipogenesis is regulated by various physiological factors, such as hormones and nuclear receptors. Insulin was found to stimulate sebaceous lipogenesis and differentiation [4,5]. Furthermore, liver X receptors (LXRs) play major roles in cholesterol homeostasis and lipid metabolism [6]. LXR activation in SZ95 sebocytes upregulates the expression of LXR target genes, including sterol regulatory-binding protein 1 (SREBP1), fatty acid synthase (FAS), and acetyl-CoA carboxylase (ACC), and increases lipid synthesis [7,8]. SREBPs are major transcription factors involved in lipid homeostasis in the liver and sebaceous glands [9,10]. The SREBP family consists of SREBP-1a, SREBP-1c, and SREBP2 [11]. SREBP1 mainly controls the transcription of genes involved in fatty acid biosynthesis, whereas SREBP2 controls genes involved in cholesterol biosynthesis [12]. SREBP-1c is the predominant isoform expressed in most human tissues and is highly expressed in the liver, white adipose tissue, skeletal muscle, adrenal gland, and brain [13].

Histone acetylation is a major epigenetic modification that reversibly alters the chromatin structure for transcriptional activation [14,15]. Histone acetylation is mediated by histone acetyltransferases (HATs) and removed by histone deacetylases (HDACs). HATs relax DNA–histone interactions, leading to transcriptional activation, whereas HDACs condense DNA–histone interactions, resulting in transcriptional repression [16]. Our previous study revealed that anacardic acid (AA), a p300 HAT inhibitor, suppressed SREBP1 expression and lipogenesis in human differentiated adipocytes by suppressing histone acetylation [17]. Hyperacetylation of H3K9 and H4K8 has been reported to activate lipogenic gene transcription in hepatocytes [18]. Conversely, inhibition of HDAC activity with sodium butyrate resulted in adipogenesis in 3T3-L1 adipocytes [19]. However, the epigenetic regulatory mechanism underlying lipogenesis in sebaceous glands remains unclear.

In humans, 11 classical histone deacetylases (HDAC1-HDAC11) and seven sirtuins (SIRT1-SIRT7) have been identified as HDACs. They are subdivided into four classes based on their functions and DNA sequence similarity. Class I is homologous to reduced potassium dependency 3 (Rpd3) in yeast and includes HDAC1, HDAC2, HDAC3, and HDAC8. Class II consists of HDAC4, HDAC5, HDAC6, HDAC7, HDAC9, and HDAC10 and shares homology with yeast Hda1. Class III HDACs (SIRT1 to SIRT7) use a nicotinamide adenine dinucleotide (NAD)-dependent mechanism. Finally, HDAC11 is the only member of class IV HDACs [20]. Classes I, II, and IV are inhibited by trichostatin A (TSA) and have a zinc-dependent active site. In contrast, class III enzymes are not affected by TSA [21]. Among the 11 classical HDACs, HDAC1 and 2, also referred to as canonical HDACs, have strong enzymatic activity towards histones [22].

In the present study, we aimed to determine the effects of histone acetylation on the lipogenic response and lipid accumulation in the immortalized human sebaceous gland cell line SZ95 and elucidate a critical epigenetic mediator regulating SREBP1 and sebaceous lipogenesis via histone acetylation. Our findings demonstrated that HDAC1 plays a critical role in reducing SREBP1 transcription, leading to decreased sebaceous lipogenesis. Hence, HDAC1 activation could be an effective therapeutic strategy for skin diseases related to excessive sebum production.

## 2. Results

### 2.1. Insulin and LXR Agonist Treatment Enhanced Lipid Accumulation and Histone Acetylation in SZ95 Sebocytes

**Figure 1:**
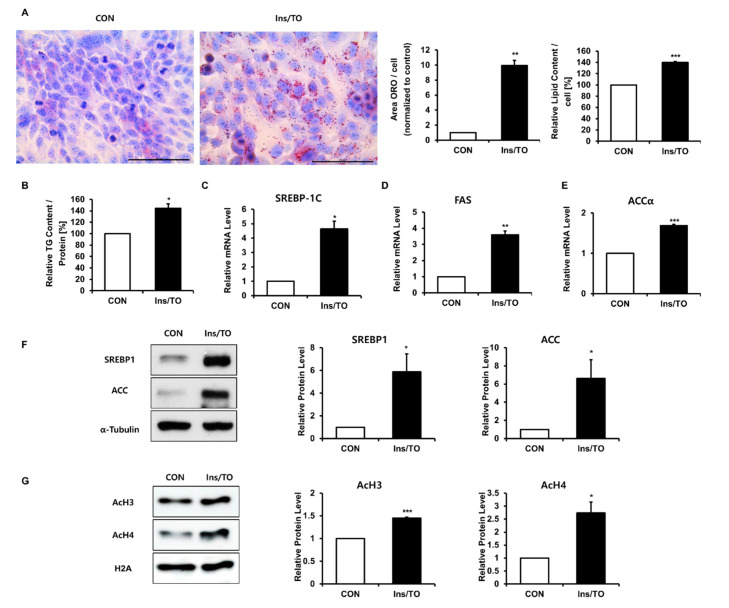
Insulin and liver X receptor (LXR) agonist treatment increased lipid synthesis and histone acetylation in SZ95 sebocytes. SZ95 sebocytes were treated with 10 µg/mL insulin and 1 µM of the LXR agonist TO901317 (Ins/TO) for 48 h or 72 h. (**A**) Intracellular lipids were detected 72 h after Oil Red O staining (left) and quantified using the Image J software (middle). Scale bar = 100 μm. After Oil Red O (ORO) staining, the lipid content was eluted into isopropanol and the optical density at 500 nm was assessed using a microplate reader (right). Values represent the mean ± SD from three independent experiments. ** *p* < 0.01, *** *p* < 0.001 vs. control. (**B**) Triglyceride (TG) content was measured at 72 h using TG analysis kits. Data represent the mean ± SD from three independent experiments. * *p* < 0.05 vs. control. (**C**–**E**) The mRNA levels of sterol regulatory element binding protein 1c (SREBP-1c), fatty acid synthase (FAS), and acetyl-CoA carboxylase α (ACCα) were analyzed using real-time PCR and normalized to the expression of 36B4 48 h after Ins/TO treatment. Data represent the mean ± SD from three independent experiments. * *p* < 0.05, ** *p* < 0.01, *** *p* < 0.001 vs. control. (**F**) SREBP1 and ACC protein expression at 48 h measured using Western blotting. Bar graphs show densitometric quantitation of SREBP1 and ACC as ratios to α -Tubulin, which was used as the loading control. Data represent the mean ± SD from three independent experiments. * *p* < 0.05 vs. control. (**G**) Histone H3 and H4 acetylation following Ins/TO treatment observed using Western blot analysis 48 h after Ins/TO treatment. Bar graphs show densitometric quantitation of histone H3 and H4 acetylation as ratios to Histone H2A, which was used as the loading control. Data represent the mean ± SD from three independent experiments (*n* = 3). * *p* < 0.05, *** *p* < 0.001 versus control.

To investigate epigenetic alterations during lipid accumulation in human SZ95 sebocytes, we first induced lipid accumulation by treating SZ95 cells with insulin (10 µg/mL) and the LXR agonist TO901317 (1 µM). Treatment with insulin and TO901317 for 72 h resulted in significant increases in lipid droplet accumulation in the cytoplasm, as visualized using Oil Red O and Nile red stainings (Figure 1A and Figure S1). As triglycerides (TG) are a major constituent of sebum [8], the TG content was also measured. Treatment with insulin and TO901317 significantly increased the TG content (Figure 1B). The mRNA and protein expression of key lipogenic genes, including SREBP1, FAS, and ACC, was also induced 48 h after insulin and TO901317 treatment (Figure 1C–F). At the same time, we extracted histones and measured histone H3 and H4 acetylation using Western blotting. We found that sebocyte lipid synthesis was associated with a significant increase in H3 and H4 acetylation to 145.1% ± 2.1% and 273.7% ± 42.5%, respectively (Figure 1G). These observations suggest that insulin- and LXR agonist-induced lipid synthesis may be closely associated with increased histone acetylation in human SZ95 sebocytes.

### 2.2. Increased Histone Acetylation Promoted Lipogenesis through SREBP1 Induction

**Figure 2:**
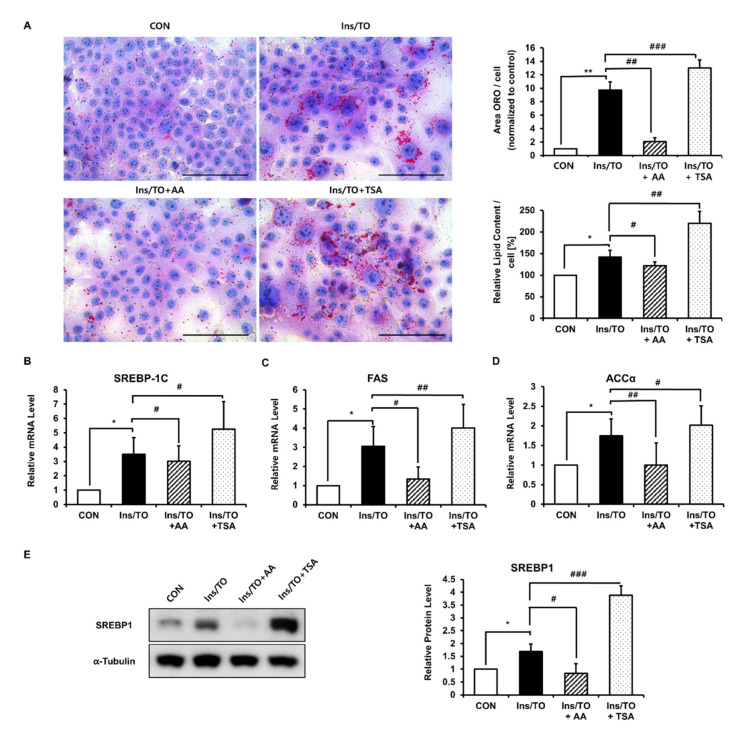
The p300 histone acetyltransferase (HAT) inhibitor anacardic acid (AA) inhibited lipogenesis through SREBP1 induction, whereas the histone deacetylase (HDAC) inhibitor trichostatin A (TSA) stimulated it. SZ95 cells were treated with 12.5 µM AA or 0.2 µM TSA in addition to insulin and liver X receptor (LXR) agonist TO901317 treatment (Ins/TO) for 48 h or 72 h. (**A**) After 72 h, Oil Red O staining was performed to analyze the amount of lipid droplets (left). Scale bar = 100 μm. Quantitative analysis of Oil Red O was performed using the Image J software. After Oil Red O staining, the lipid content was eluted into isopropanol, and the optical density at 500 nm was determined using a microplate reader. Values represent the mean ± SD from three independent experiments. * *p* < 0.05, ** *p* < 0.01 vs. control; # *p* < 0.05, ## *p* < 0.01, ### *p* < 0.001 vs. SZ95 cells treated with Ins/TO. (**B**–**D**) The expression of sterol regulatory element binding protein 1c (SREBP-1c), FAS, and ACCα mRNA was quantified by real-time PCR 48 h after AA or TSA treatment and normalized to the respective 36B4 as a control. Values represent the mean ± SD from three independent experiments. * *p* < 0.05 vs. control; # *p* < 0.05, ## *p* < 0.01 vs. SZ95 cells treated with Ins/TO. (**E**) SREBP1 protein expression was measured 48 h after treatment by Western blotting. Bar graphs show densitometric quantitation of SREBP1 as ratios to α -Tubulin, which was used as the loading control. Data represent the mean ± SD of four independent experiments (*n* = 4). * *p* < 0.05 vs. control; # *p* < 0.05, ### *p* < 0.001 vs. sebocytes treated with Ins/TO.

Next, we examined whether increased histone acetylation might promote insulin- and LXR agonist-induced sebaceous lipogenesis using a HAT inhibitor or an HDAC inhibitor. Treatment with the HAT inhibitor AA (12.5 µM), insulin, and the LXR agonist for 72 h significantly prevented lipid accumulation. In contrast, treatment with the HDAC inhibitor TSA (0.2 µM) for 72 h induced lipid accumulation in SZ95 cells (Figure 2A and Figure S2). Furthermore, treatment with AA led to a significant decrease in the mRNA expression of SREBP-1c, FAS, and ACCα, whereas treatment with TSA led to significant increases in their mRNA levels at 48 h compared to treatment with insulin and LXR agonist (Figure 2B–D). As SREBP1 is the master regulator of sebaceous lipogenesis [23] and regulates the expression of ACC and FAS, we next examined SREBP1 protein expression after AA or TSA treatment. At 48 h, SREBP1 protein expression was dramatically decreased by AA treatment and increased by TSA treatment compared to insulin and LXR agonist treatment (Figure 2E). Taken together, these data showed that modulation of histone acetylation using a HAT or an HDAC inhibitor altered SREBP1 expression and sebaceous lipogenesis, indicating that increased histone acetylation may play a major role in sebaceous lipogenesis.

### 2.3. Insulin and LXR Agonist Treatment Decreased Overall HDAC Activity, and Specifically HDAC1 and HDAC2 Protein Expression

**Figure 3:**
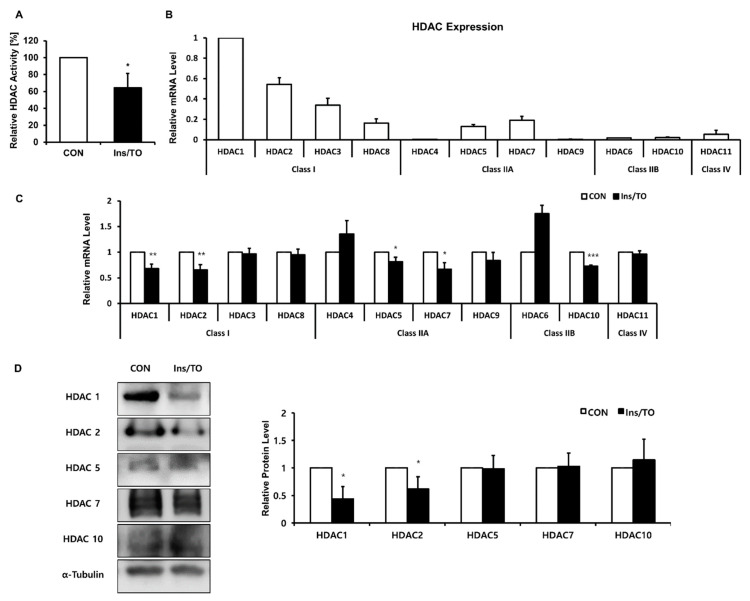
Decreased histone deacetylase (HDAC) activity and HDAC1 and HDAC2 expression levels during lipogenesis in SZ95 cells. (**A**) Total HDAC activity of SZ95 cells analyzed using an HDAC assay kit 48 h after insulin and liver X receptor (LXR) agonist (Ins/TO) treatment. (**B**) Relative HDAC mRNA expression in SZ95 cells analyzed using quantitative real-time PCR and normalized to that of HDAC1. (**C**) HDAC mRNA expression analyzed using quantitative real-time PCR and normalized to that of 36B4. (**D**) HDAC protein expression measured using Western blotting 48 h after Ins/TO treatment. Bar graphs show densitometric quantitation of HDACs as ratios to α-Tubulin, which was used as the loading control. Data represent the mean ± SD from three independent experiments (*n* = 3). * *p* < 0.05, ** *p* < 0.01, *** *p* < 0.001 vs. control.

To address the role of HDACs in the increased histone acetylation involved in sebaceous lipogenesis, the overall HDAC activity was measured 48 h after treatment with insulin and the LXR agonist TO901317. The total HDAC activity of sebocytes treated with insulin and TO901317 significantly decreased up to 64.2 ± 16.9%, compared to that of vehicle-treated sebocytes (Figure 3A). Next, we explored to discover the key HDAC in the regulation of sebocyte lipogenesis. To determine different expression levels among HDACs, we compared the relative expression of HDAC isoforms in SZ95 cells. HDAC1 was the most highly expressed HDAC isoform (Figure 3B). Subsequently, we analyzed the mRNA expression of the 11 classical HDAC family members after treatment with insulin and the LXR agonist and found that the mRNA expression of HDAC1, HDAC2, HDAC5, HDAC7, and HDAC10 was significantly decreased at 48 h (Figure 3C). To investigate whether the protein expression of these HDACs coincided with their mRNA expression, we validated the protein expression of HDAC1, HDAC2, HDAC5, HDAC7, and HDAC10 48 h following treatment with insulin and TO901317. HDAC1 and HDAC2 were the only two HDAC isoforms that exhibited significant decreases in their protein expression 48 h after treatment with insulin and TO901317 (Figure 3D). These results suggest that HDAC1 might play an important role in increased histone acetylation, leading to sebaceous lipogenesis.

### 2.4. Decreased HDAC1 Is Crucial for Insulin and LXR Agonist-Induced Lipid Synthesis in SZ95 Sebocytes

**Figure 4:**
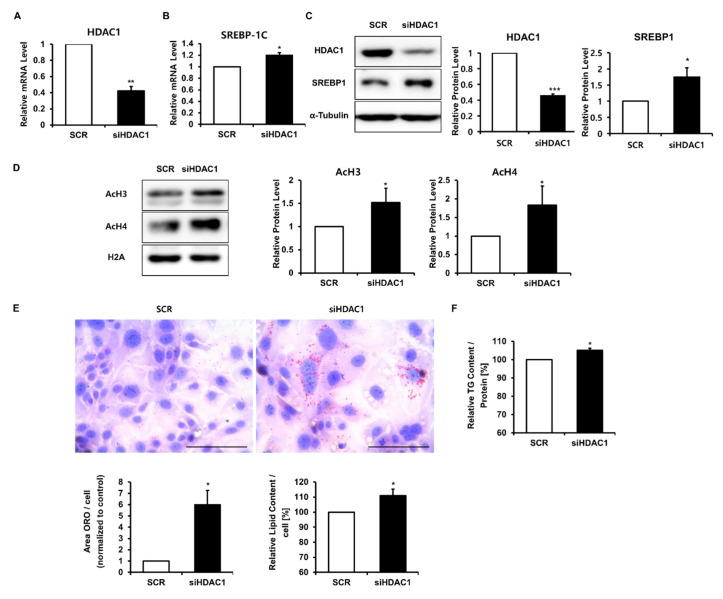
Histone deacetylase 1 (HDAC1) knockdown induced sterol regulatory element binding protein 1c (SREBP-1c) expression and lipogenesis in SZ95 cells. SZ95 cells were transfected with 100 nM negative control scrambled siRNA (SCR) or HDAC1 siRNA (siHDAC1) for 6 h and the medium was replaced with 10% FBS. After 48 h, (**A**,**B**) HDAC1 and SREBP-1c mRNA expression was measured using real-time PCR and (**C**) HDAC1 and SREBP1 protein expression was measured using Western blotting. α-Tubulin served as a loading control. (**D**) Histone H3 and H4 acetylation observed using Western blot analysis 48 h after HDAC1 knockdown. Histone H2A served as a loading control. (**E**) Lipid contents were visualized by Oil Red O staining, quantified using the Image J software (left), and eluted into isopropanol at 72 h. The optical density at 500 nm was determined using a microplate reader (right). Scale bar = 100 μm. (**F**) Triglyceride (TG) content analysis using a TG assay kit performed 72 h after HDAC1 knockdown. Data represent the mean ± SD of three independent experiments (*n* = 3). * *p* < 0.05, ** *p* < 0.01, *** *p* < 0.001 vs. scramble siRNA-transfected control.

To determine whether decreased HDAC1 expression is sufficient to induce SREBP1 expression and increased lipid synthesis, we transfected SZ95 cells with small interfering RNAs (siRNAs) targeting HDAC1 expression (HDAC1 siRNA) or scrambled siRNA. SZ95 cells transfected with HDAC1 siRNA showed significant increases in both SREBP1 mRNA and protein expression compared to SZ95 cells transfected with scrambled siRNA 48 h after siRNA transfection (Figure 4A–C). Histone acetylation also increased 24 h after HDAC1 knockdown (Figure 4D). Oil Red O and Nile red stainings revealed that HDAC1 knockdown enhanced lipid synthesis at 72 h (Figure 4E and Figure S3). The TG content was also significantly increased 72 h after HDAC1 knockdown (Figure 4F).

**Figure 5:**
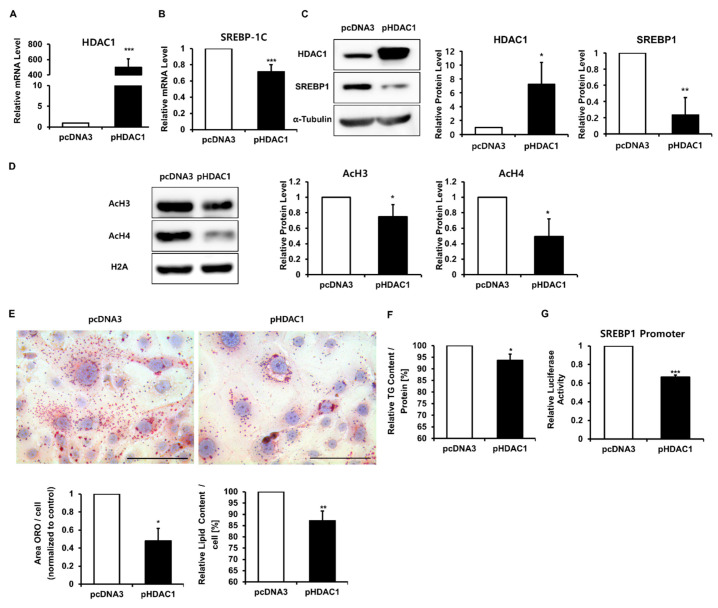
Histone deacetylase 1 (HDAC1) suppressed sterol regulatory element binding protein 1c (SREBP-1c) transcription and lipogenesis in SZ95. SZ95 cells were transfected with the pcDNA3 control vector or HDAC1 plasmid DNA for 6 h and the medium was replaced with medium containing 2% FBS, insulin, and the liver X receptor (LXR) agonist TO901317. Cells were harvested at 48 h for mRNA, protein, and histone protein analyses. (**A**,**B**) HDAC1 and SREBP-1c mRNA levels analyzed using real-time PCR. (**C**) HDAC1 and SREBP1 protein levels measured using Western blotting. α-Tubulin served as a loading control. (**D**) Histone H3 and H4 acetylation observed using Western blot analysis. Histone H2A served as a loading control. (**E**) Oil Red O staining was performed to analyze the amount of lipid droplets at 72 h. Scale bar = 100 μm. Quantitative analysis of Oil Red O was performed using the Image J software (left). The lipid content was eluted into isopropanol, and the optical density at 500 nm was determined using a microplate reader (right). (**F**) Triglyceride (TG) content analysis using a TG assay kit performed at 72 h. (**G**) SREBP1 promoter activity was measured using a luciferase assay 24 h after pcDNA3 or HDAC1 plasmid transfection. Data represent the mean ± SD of four independent experiments (*n* = 3). * *p* < 0.05, ** *p* < 0.01, *** *p* < 0.001 vs. pcDNA3-transfected control.

To further assess whether decreased HDAC1 is necessary for insulin and LXR agonist-induced lipogenesis, we transfected SZ95 cells treated with insulin and the LXR agonist with the HDAC1-pcDNA3 plasmid or pcDNA3-mock plasmid. Overexpression of HDAC1 significantly decreased the mRNA and protein levels of SREBP1 48 h after transfection (Figure 5A–C). Histone acetylation also decreased 24 h after HDAC1 overexpression (Figure 5D). Furthermore, HDAC1 overexpression reduced lipid accumulation and the TG content in SZ95 cells at 72 h (Figure 5E,F and Figure S4). To further explore whether HDAC1 regulated SREBP1 transcription, we analyzed SREBP1 promoter activity 24 h after transfection with the pcDNA3-mock control or HDAC1-pcDNA3 plasmid in the presence of insulin and the LXR agonist TO901317. Overexpression of HDAC1 resulted in a significant decrease in SREBP1 promoter activity (Figure 5G). These results imply that decreased HDAC1 is necessary and sufficient for insulin and LXR agonist-induced sebaceous lipogenesis.

### 2.5. The HDAC1 and SREBP1 Expression Levels Were Inversely Correlated in Human Sebaceous Glands

**Figure 6:**
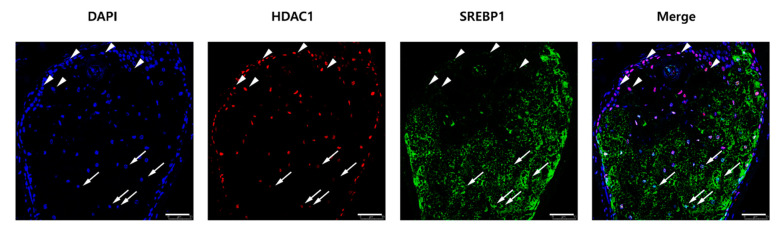
Histone deacetylase 1 (HDAC1) and sterol regulatory element binding protein 1 (SREBP1) expression levels were inversely correlated in normal human sebocytes. Sebaceous glands in normal human skin tissues were subjected to double immunofluorescence staining for HDAC1 (red) and SREBP1 (green). Arrows show sebocytes with low HDAC1 and high SREBP1, whereas arrowheads show sebocytes with high HDAC1 and low SREBP1. Nuclei were counterstained with DAPI. Scale bar = 50 μm; ×300 magnification.

Finally, we performed double immunofluorescence staining for HDAC1 and SREBP1 in normal sebaceous glands of human skin (*n* = 3). Co-staining of SREBP1 and HDAC1 revealed that their expression patterns were inversely correlated (Figure 6). HDAC1 was most strongly localized in the basal parabasal zones of sebaceous glands. In contrast, SREBP1 was highly expressed in the central region of the sebaceous glands. Hence, proliferating sebocytes in the basal-parabasal region showed high expression of HDAC1, whereas lipid-synthesizing sebocytes in the central region showed high expression of SREBP1. More specifically, sebocytes with lower expression of HDAC1 showed higher expression of SREBP1 (Figure 6, arrow), whereas sebocytes with higher expression of HDAC1 showed lower expression of SREBP1 (Figure 6, arrowhead). These in vivo findings support the hypothesis that HDAC1 negatively regulates SREBP1 expression and lipid accumulation in human sebaceous glands.

## 3. Discussion

Histone acetylation plays a predominant role in lipid biosynthesis and differentiation in several cell types, including hepatocytes and adipocytes [19,24,25]. Our previous study showed that histone acetylation regulates SREBP1 transcription, leading to lipid accumulation in primary human adipocytes [17]. Various key lipogenic genes in adipocytes have also been identified to mediate sebocyte lipogenesis and differentiation. Peroxisome proliferator-activated receptors (PPARs) increase sebaceous lipid production and differentiation [26]. Lipogenic factors in adipocytes such as galectin-12, resistin, SREBP1, and stearoyl CoA desaturase (SCD) are also detected in human sebaceous glands [27]. These similarities in lipogenesis between adipocytes and sebocytes led to the hypothesis that histone acetylation might also be pivotal in regulating human sebum production. However, little is known about the role of histone acetylation in sebocyte lipogenesis.

In the present study, we found that lipid synthesis in sebocytes was associated with a significant increase in histone acetylation under physiological conditions. Consistent with our results, a recent study revealed that histone acetylation was also increased under inflammatory conditions with short-chain fatty acids from *Cutibacterium acnes* in human sebocytes [28]. Therefore, modulation of histone acetylation and the resultant regulation of excess sebum production and inflammatory responses could be an effective therapeutic strategy against acne vulgaris. Furthermore, we demonstrated that HDAC1 might play an important role in regulating histone acetylation and sebaceous lipogenesis both in vitro and in vivo. To our knowledge, this is the first evidence that underscores the important role of HDAC1 in sebaceous lipogenesis and the inverse correlation between HDAC1 and SREBP1 expression. In 3T3-L1 adipocytes, the expression of HDAC1, HDAC2, HDAC5, and HDAC6 was substantially reduced during lipogenesis, and HDAC1 overexpression inhibited lipid accumulation [19]. In contrast, HDAC5 expression was reduced in the liver of obese mice, and HDAC5 inhibited hepatic lipogenic gene expression by suppressing the transcriptional activity of LXR [29]. Taken together, these studies suggest that specific types of HDACs regulate genes associated with lipid metabolism in different cells and organs.

We focused on HDAC1, not HDAC2, for the following reasons. First, HDAC1 was the most highly expressed HDAC isoform in SZ95 sebocytes. Second, HDAC1 showed a more dramatic decrease in protein expression than HDAC2 during sebaceous lipogenesis, despite similar decreases in their mRNA levels. HDAC1 and HDAC2 are highly similar enzymes that bind to each other, forming co-repressor complexes [30]. However, Yamaguchi et al. revealed that approximately 40% of HDAC1 exists independently of HDAC2. Furthermore, transcriptome analysis and histone modification assays demonstrated that HDAC1 and HDAC2 have partly overlapping roles [31]. For these reasons, HDAC1 might have a distinct role from HDAC2 in regulating sebaceous lipogenesis.

In conclusion, our study uncovered a significant epigenetic mechanism involved in sebum production in human sebocytes. Since HDAC1 might be a key HDAC regulating sebaceous lipogenesis, HDAC1 activators may be helpful in the treatment of skin diseases related to excessive sebum production.

## 4. Materials and Methods

### 4.1. Cell Culture

The immortalized human sebaceous gland cell line SZ95 [32] was maintained in DMEM/Ham’s F12 medium (3:1) containing 10% fetal bovine serum (Welgene, Daegu, South Korea), 1% penicillin streptomycin (Gibco, Rockville, MD, USA), and 5 ng/mL human epidermal growth factor (Sigma, St Louis, MO, USA) in a humidified 37 °C incubator with 5% CO_2_. The medium was replaced every 2–3 days.

### 4.2. Chemicals and Reagents

Insulin, TO901317, AA, and TSA were purchased from Sigma Aldrich (St. Louis, MO, USA). Anti-ACC, acetyl-histone H3, acetyl-histone H4, histone H2A, and HDAC5 antibodies were purchased from Cell Signaling Technology (Danvers, MA, USA). Anti-SREBP1, α-Tubulin, HDAC1, HDAC2, HDAC7, and HDAC10 antibodies were obtained from Santa Cruz Biotechnology (Santa Cruz, CA, USA).

### 4.3. Oil Red O Staining

SZ95 sebocytes were washed with phosphate-buffered saline (PBS) and fixed in 10% formaldehyde for 10 min. Fixed cells were stained for 30 min with filtered 0.7% Oil Red O solution (Sigma, St Louis, MO, USA) in propylene glycol. Stained cells were washed with distilled water, counterstained with hematoxylin, and visualized using microscopy. For quantitative analysis of Oil Red O staining, the area of lipid droplets was measured using the Image J software and normalized by the number of nuclei. For quantitative detection of intracellular lipids, Oil Red O was eluted by incubating cells with isopropanol for 10 min. Supernatant Oil Red O levels were determined by measuring the optical density at 500 nm using a microplate reader. To calculate the lipid amount per cell, the optical density value was normalized to the cell count and measured using an ADAM-MC automatic cell counter and the ADAM-MC software (Bulldog Bio, Portsmouth, NH, USA).

### 4.4. Nile Red Staining

For Nile red staining, cells were washed in PBS and fixed with 4% formaldehyde at room temperature for 15 min. Fixed cells were stained with 1 μg/mL Nile red solution (Sigma, St. Louis, MO, USA) for 15 min. Images of stained cells were acquired using confocal microscopy. Fluorescence intensity was quantified in the Image J software and normalized by the number of cells.

### 4.5. Determination of the TG Content

The TG content was determined based on a fluorescent enzymatic method using commercially available kits (Asan Pharmaceutical Co. Ltd., Seoul, Korea) and normalized to the protein content measured using the Bradford method (Bio-Rad, Hercules, CA, USA).

### 4.6. siRNA Transfection

HDAC1 gene silencing was performed using small interfering RNAs (siRNAs). Negative control siRNA (AccuTarget™ Negative control siRNA) and HDAC1 siRNA were obtained from Bioneer (Daejeon, Republic of Korea). The HDAC1 siRNA sequence was as follows: sense 5′-GAGUCAAAACAGAGGAUGA dTdT-3′, antisense 5′-UCAUCCUCUGUUUUG ACUCdTdT-3′. When SZ95 cells reached 60% confluence, 100 pM siRNAs were transfected using jetPRIME^®^ (Polyplus-transfection^®^ S, Illkirch, France) and incubated for 48 h or 72 h.

### 4.7. Plasmid Constructs, Transient Transfection, and Luciferase Reporter Assay

HDAC1 overexpression was performed by transiently transfecting 80% confluent SZ95 cells with 1 µg pcDNA3 or HDAC1-Flag plasmid (Addgene, Cambridge, MA, USA), using jetOPTIMUS^®^ (Polyplus-transfection^®^ SA, Illkirch, France) for 6 h. The medium was replaced with DMEM/Ham’s F12 medium (3:1) containing 2% FBS, insulin (10 µg/mL), and TO901317 (1 µM), and the cells were harvested after 48 h for mRNA and protein extraction or after 72 h for lipid analysis.

The human SREBP1 promoter/luciferase plasmid (pGL3 Basic-pSREBP1) contained the firefly luciferase gene under the transcriptional control of the human SREBP1 promoter in the pGL3 basic reporter vector (Promega, Madison, WI, USA). For luciferase assays, 80% confluent SZ95 cells were transiently co-transfected with SREBP1-Luc pcDNA3 or the HDAC1-flag plasmid using jetPRIME^®^ (Polyplus-transfection^®^ SA, Illkirch, France). After 24 h, the cells were lysed and luciferase activity was measured. The pRL-TK plasmid was used as an internal control for transfection efficiency. Luciferase activity was assayed, and reporter activity was normalized to that of cells co-transfected with pcDNA3.

### 4.8. Histone Extraction and Western Blotting

Histone proteins were extracted from SZ95 cells using a histone extraction kit (Abcam, Cambridge, UK) according to the manufacturer’s instructions. A total of 20 µg of histone proteins or 40 µg of protein extracts were resolved on SDS-polyacrylamide gels and then transferred onto nitrocellulose and polyvinylidene fluoride membranes, respectively. After blocking for 1 h in 5% skim milk, the membrane was incubated overnight with primary antibodies (1:1000) at 4 °C. The membranes were then washed and incubated with horseradish peroxidase-linked secondary antibody (1:10,000) for 1 h at room temperature. Immunoreactive proteins were detected using the ECL substrate from Biomax (Seoul, Korea).

### 4.9. Immunofluorescence Staining

Human skin tissues were fixed in 10% formalin for 24 h and embedded in paraffin wax before they were cut into 4 µm sections. Following the standard procedure, the tissue was blocked for 30 min at room temperature using a blocking solution and incubated overnight in a humidified chamber at 4 °C with the following primary antibodies: polyclonal anti-HDAC1 (Santa Cruz Biotechnology, Santa Cruz, California, USA) 1:500 in TRS pH 9.0, and polyclonal anti-SREBP1 (Santa Cruz Biotechnology, Santa Cruz, CA, USA) 1:500 in TRS pH 9.0. The bound primary antibodies were detected using streptavidin-conjugated Alexa 488 and Alexa 594 secondary antibodies (Invitrogen, Carlsbad, CA, USA). Nuclei were counterstained with 4′,6-diamidino-2-pheylindole (DAPI). The fluorescent images were obtained using a confocal microscope.

### 4.10. Quantitative Real-Time Polymerase Chain Reaction (PCR)

Total RNA was isolated from human SZ95 sebocytes using RNAiso Plus (Takara Bio Inc., Shiga, Japan) and then reverse transcribed into cDNA using the First Strand cDNA Synthesis Kit (MBI Fermentas, Vilnius, Lithuania) according to the manufacturer’s instructions. cDNA was amplified using a 7500 Real-time PCR System (Applied Biosystems, Foster City, CA, USA) and SYBR Premix Ex Taq (Takara Bio Inc., Shiga, Japan) according to the manufacturer’s instructions. The 2-∆∆Ct method was used to calculate the fold change in the expression level of the target gene from its threshold cycle values, which were normalized to those of 36B4. The primer sequences used are listed in Table 1.

### 4.11. Histone Deacetylase Activity Assay

Global HDAC enzymatic activity was measured using an HDAC assay kit (Upstate Biotech, NY, USA) according to the manufacturer’s protocol. Briefly, 20 µg of total cell extracts from SZ95 cells were incubated with the HDAC assay substrate for 60 min at 37 °C, allowing deacetylation of the colorimetric substrate. Then, the activator solution was added to release the colorimetric molecules from the deacetylated substrates. The absorbance was measured at 405 nm using a plate reader.

### 4.12. Human Skin Samples

Human volunteers (*n* = 3) provided the skin samples, which were obtained using a punch biopsy. All procedures involving human subjects were approved by the Seoul National University Institutional Review Board (IRB number: 1908-052-1055), and the volunteers provided written informed consent. The study was conducted in accordance with the principles of the Declaration of Helsinki.

### 4.13. Statistical Analysis

All statistical analyses were performed using the Microsoft Excel 2015 software. Statistical significance was determined using the Student’s *t*-test. Results are presented as the mean ± SD. Two-tailed *p*-values were calculated, and a *p*-value of <0.05 was considered statistically significant.

## Figures and Tables

**Table 1:** Primer sequences for quantitative real-time PCR.

Gene	Primers (5′ - 3′)	
	Forward	Reverse
36B4	TCGACAATGGCAGCATCTAC	TGATGCAACAGTTGGGTAGC
SREBP-1c	GCCATGGATTGCACTTT	CAAGAGAGGAGCTCAATG
ACCα	ATGCTGACCGAGAAAGCA	TGCGGATTTGCTTGAGGA
FAS	CCGAGGAACTCCCCTCAT	GCCAGCGTCTTCCACACT
HDAC1	ATCTATCGCCCTCACAAAGC	AATCTCTGCATCTGCTTGCT
HDAC2	TGCTACTACTACGACGGTGA	AGTGGCTTTATGGGGCCTA
HDAC3	GAGAGTCAGCCCCACCAATA	GTTGTTCAGCTGGGTTGCTC
HDAC4	GAG AGA CTC ACC CTT CCC G	CCG GTC TGC ACC AAC CAA G
HDAC5	ACAGCATGACCCCTGACAAGG	GCT CCT GCT GCC GCT TGG
HDAC6	ATCTGGCGGAGTGGAAGAA	AAGTGACACTGGAGTCCTGA
HDAC7	CTCACTGTCAGCCCCAGAG	CTGGTGCTTCAGCATGACC
HDAC8	AAACGGGCCAGTATGGTG	CTGACCTTCTGGAGATGCTG
HDAC9	CAACAAAACCCTAGCAGCCT	GCCCACAGGAACTTCTGACT
HDAC10	CACTAGCGAGGGCGTTTG	GGGTCGTCCCAGAGCA
HDAC11	GGATGCTACACACAACCCA	CCCATTTTCCGGCATCAAAG

## Data Availability

The data that support the findings of this study are available from the corresponding author upon reasonable request.

## Supplementary Materials

Supplementary materials can be found at https://www.mdpi.com/article/10.3390/ijms22094477/s1.
