# Supplementary material for: Histone Deacetylase 1 Reduces Lipogenesis by Suppressing SREBP1 Transcription in Human Sebocyte Cell Line SZ95
Source: Int J Mol Sci. 2021 Apr 25;22(9):4477. doi: 10.3390/ijms22094477 (PMC8123291; doi:10.3390/ijms22094477)

Histone deacetylase 1 reduces lipogenesis by suppressing SREBP1 transcription in human sebocyte cell line SZ95

Hye Sun Shin [1,2,3], Yuri Lee [1,2,3], Mi Hee Shin [1,3], Soo Ick Cho[1,3], Christos C. Zouboulis [4], Min-Kyoung Kim [1,3,*] , Dong Hun Lee [1,3,*] and Jin Ho Chung [1,2,3,5,*]

[1] Department of Dermatology, Seoul National University College of Medicine, Seoul 03080, Korea; shs6211@snu.ac.kr (H.S.S.); lepommier@snu.ac.kr (Y.L.); fiona1@hanmail.net (M.H.S.); chlroe@hotmail.com (S.I.C.)

[2] Department of Biomedical Sciences, Seoul National University Graduate School, Seoul 03080, Korea

[3] Institute of Human-Environment Interface Biology, Medical Research Center, Seoul National University, Seoul 03080, Korea

[4] Departments of Dermatology, Venereology, Allergology and Immunology, Dessau Medical Center, Brandenburg Medical School Theodor Fontane and Faculty of Health Sciences Brandenburg, Dessau 06847, Germany; christos.zouboulis@klinikum-dessau.de (C.C.Z.)

[5] Institute on Aging, Seoul National University, Seoul 03080, Korea

* Correspondence: 82772@snuh.org (M.-K.K.); ivymed27@snu.ac.kr (D.H.L.); jhchung@snu.ac.kr (J.H.C.)

Supplementary results

Supplementary Figure S1. Insulin and LXR agonist treatment enhanced lipid accumulation in SZ95 sebocytes. Insulin and liver X receptor (LXR) agonist treatment increased lipid synthesis in SZ95 sebocytes. SZ95 sebocytes were treated with 10 µg/ml insulin and 1 µM of the LXR agonist TO901317 (Ins/TO) for 72 h. (A) Intracellular lipids were detected by Nile red staining using a laser scanning confocal microscopy. (B) Fluorescence intensity was quantified in Image J software and normalized by the number of cells. Intracellular lipids are shown in red. Scale bar = 25 µm. Values represent the mean ± SD from three independent experiments. *P<0.05 vs control

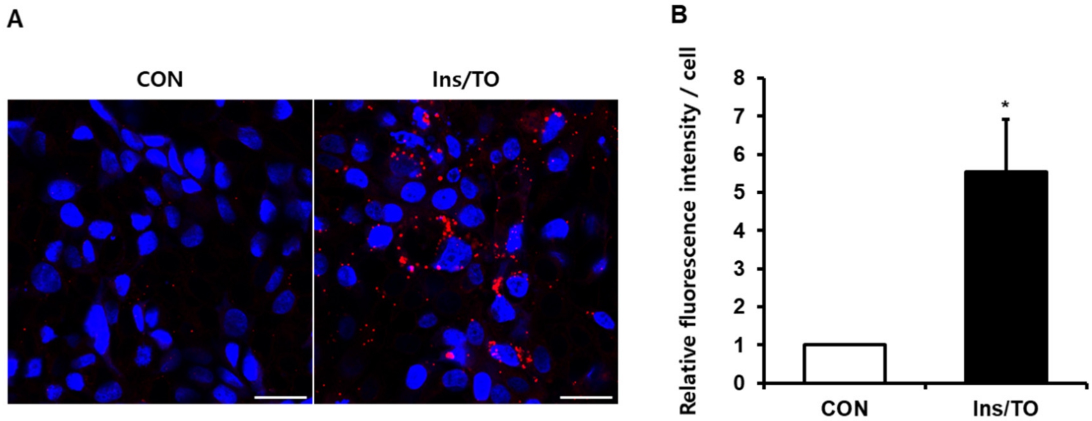

Supplementary Figure S2. The p300 histone acetyltransferase inhibitor anacardic acid (AA) inhibited lipid accumulation, while the histone deacetylase inhibitor trichostatin A (TSA) stimulated it. SZ95 cells were treated with 12.5 μM AA or 0.2 μM TSA in addition to insulin and LXR agonist TO901317 treatment (Ins/TO) for 72 h. (A) Intracellular lipids were detected by Nile red staining using a laser scanning confocal microscopy. (B) Fluorescence intensity was quantified in Image J software and normalized by the number of cells. Intracellular lipids are shown in red. Scale bar = 25 μm. Values represent the mean ± SD from three independent experiments. Values represent the mean ± SD from three independent experiments. *P<0.05 vs control; #P<0.05, ##P<0.01 vs SZ95 cells treated with Ins/TO.

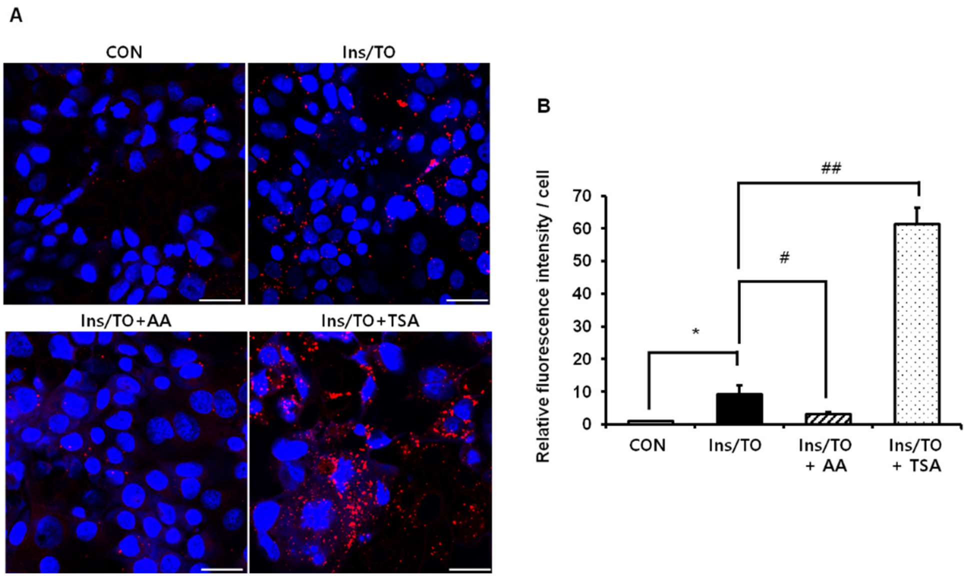

Supplementary Figure S3. Histone deacetylase 1 (HDAC1) knockdown induced lipogenesis in SZ95 cells. SZ95 cells were transfected with 100nM negative control scrambled siRNA (SCR) or HDAC1 siRNA (siHDAC1) for 6 h and the medium was replaced with 10% FBS. (A) Intracellular lipids were detected by Nile red staining using a laser scanning confocal microscopy. (B) Fluorescence intensity was quantified in Image J software and normalized by the number of cells. Intracellular lipids are shown in red. Scale bar = 25 µm. Values represent the mean ± SD from three independent experiments. *P<0.05 vs scramble siRNA-transfected control.

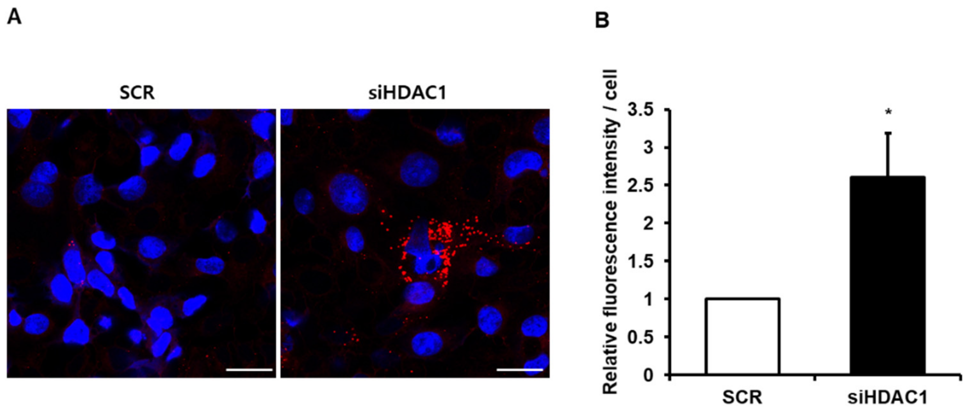

Supplementary Figure S4. Histone deacetylase 1 (HDAC1) suppressed lipogenesis in SZ95. SZ95 cells were transfected with the pcDNA3 control vector or HDAC1 plasmid DNA for 6 h and the medium was replaced with medium containing 2% FBS, insulin, and the liver X receptor (LXR) agonist TO901317. (A) Intracellular lipids were detected by Nile red staining using a laser scanning confocal microscopy. (B) Fluorescence intensity was quantified in Image J software and normalized by the number of cells. Intracellular lipids are shown in red. Scale bar = 25 μm. Values represent the mean ± SD from three independent experiments. **P<0.01 vs pcDNA3-transfected control.

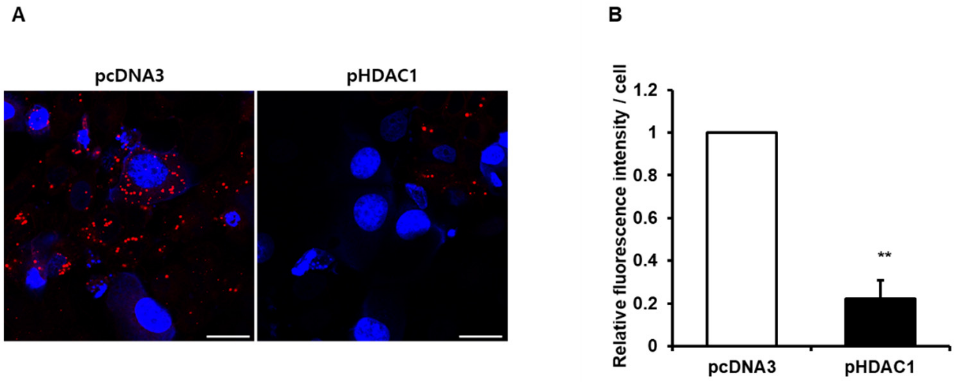

Supplement: Supplementary file 1 [file ijms-22-04477-s001.zip › ijms-1157565-supplementary.pdf]
